# Classification of the Effort Index and Biomechanical Overload in Natural Trails of UNESCO Global Geoparks—A Network Perspective of Trails of the Araripe UGG (NE Brazil)

**DOI:** 10.3390/ijerph192114297

**Published:** 2022-11-01

**Authors:** Eduardo S. Guimarães, Artur A. Sá, Rafael C. Soares, Paulo Felipe R. Bandeira, Helena Moreira, Jaqueliny R. S. Guimarães, Francisco do Ó de Lima Júnior, Ronaldo C. D. Gabriel

**Affiliations:** 1 Department of Sport Sciences, Exercise and Health, UTAD—University of Trás-os-Montes and Alto Douro, Quinta de Prados, 5000-801 Vila Real, Portugal; 2 Department of Physical Education, URCA—Universidade Regional do Cariri, Araripe UNESCO Global Geopark, Rua Cel. Antônio Luiz 1161, Crato 63105-000, CE, Brazil; 3 Department of Geology, UTAD—University of Trás-os-Montes and Alto Douro, Quinta de Prados, 5000-801 Vila Real, Portugal; 4 Araripe UNESCO Global Geopark, URCA—Universidade Regional do Cariri, Rua Carolino Sucupira, Crato 63105-000, CE, Brazil; 5 Motor Evaluation Study, Application and Research Group—GEAPAM, URCA—Universidade Regional do Cariri, Rua Cel. Antônio Luis 1161, Crato 63105-000, CE, Brazil; 6 Department of Sport Sciences, Exercise and Health, Research Center in Sports Sciences, Health Sciences and Human Development (CIDESD) and Centre for the Research and Technology of Agro-Environmental and Biological Sciences (CITAB), University of Trás-os-Montes and Alto Douro, Quinta de Prados, 5000-801 Vila Real, Portugal; 7 Department of Medicine, URCA—Universidade Regional do Cariri, Rua Cel. Antônio Luiz 1161, Crato 63105-000, CE, Brazil; 8 Department of Economics, URCA—Universidade Regional do Cariri, Araripe UNESCO Global Geopark, Rua Cel. Antônio Luiz 1161, Crato 63105-000, CE, Brazil; 9 Department of Sport Sciences, Exercise and Health, Centre for the Research and Technology in Agro-Environmental and Biological Sciences (CITAB), University of Trás-os-Montes and Alto Douro, Quinta de Prados, 5000-801 Vila Real, Portugal

**Keywords:** geotourism, health provision, ecosystem service, green exercise, health lifestyle, sustainable development, network analysis

## Abstract

Natural trails in UNESCO Global Geopark territories show strong salutogenic, inclusive and interactive characteristics as potentials and opportunities for ecosystem health. It is essential to provide information to inform the hiker as to the characteristics of the environment and the attractions and challenges of the route. Based on a network analysis methodology we aimed to identify the indicators of centrality and strength of connection in order to classify the effort index and biomechanical overload of the Araripe UNESCO Global Geopark trails in Brazil. The results showed strong connection and centrality of the variables related to the biomechanical overload in the effort index. In the trail of Pontal de Cruz the altimetric variation and the surface of the ground are highlighted in the biomechanical overload that presented a horizontal course equivalent 2.6 times larger than the presented distance. In Sítio Fundão trail, the surface of the ground also stood out, increasing the exposure in 36% of the presented distance. On the Missão Velha Waterfall trail, the variable that stood out was the biomechanical overload on the knee, equivalent to a horizontal increase of 28% of the measured distance. The methodology presented sought to optimise the mapping, management and consolidation of a network of natural trails aggregated to a high geotouristic, scientific, educational, cultural and well-being potential as presented in the Araripe UGG territory.

## 1. Introduction

The global COVID-19 pandemic that ravages us leaves several marks and lessons in various contexts and settings. One of them is directly related to social confinement as quarantine measures, in order to decrease the potential for contagion, which was adopted on a global scale at the peak of the pandemic [1,2,3,4,5]. In this scenario, the simple act of walking on a trail and connecting with nature and the diversity of the ecosystem proved to be even more necessary for our full health [6,7,8,9,10,11]. The act of “thinking” nature, as the first access channel, in many situations, was the only way we could benefit from this connection [12,13,14,15]. The curious thing is that whenever we are encouraged to abstract, to relax, to meditate or to control our emotions, we seek to transport our thoughts to natural environments. A waterfall, the sea, a green meadow, a stream, a pond, a forest, a trail... We recall our memories of places where we have already been, or that we have seen in a film or an image [12,13,14]. The very spiritual concept of “paradise” is linked to a natural environment and never to an urban, arid or industrialized environment [14,16,17,18,19].

Despite the romanticization of the issue, it is emphasized by the increasingly emerging need for equitable global policies to integrate and order urban areas with green spaces, natural corridors and trails, sustainable mobility, restriction in fossil gas emissions, incentives for zero carbon and renewable energy programs, and sustainable use of ecosystem resources. These perspectives are no longer a utopian reality, but the only possible one, in counterpoint to the status quo mainly of the most industrialized countries and their consequent contribution to the climate changes that devastate the whole planet with potentially irreversible and catastrophic consequences [20,21,22,23,24,25,26,27,28]. In the most critical period of social distancing and with the relocation of many jobs to working from home [29,30,31,32], our need to move and enjoy the intangible benefits of natural environments has become evident [6,7,8,9,10]. In this pandemic perspective of restriction to “doses of nature” [11,12,13], the offer of active itineraries and experiences linked to diverse natural environments demonstrates a significant potential for the resumption of tourism in a sustainable character, considering that this was one of the most impacted sectors in the economic context as a result of the restrictions of social mobility on a global scale [33,34,35,36,37,38].

### 1.1. UNESCO Global Geopark Program (UGGp)

The UGGp is characterized by presenting an aggregative and dynamic way of managing a living territory from the conception and holistic articulation of the natural and cultural heritage and having geosites as places of relevant scientific, educational and touristic interest, with emphasis especially on the strategies of geoconservation, geotourism and geoeducation. However, it is not limited to the geological identity and the characterization of these geosites, as it is in dialogue with the whole territory, articulating and gathering different social sectors as the public, the private and the organized society, in order to discuss, propose and contribute with strategies to safeguard, promote and implement initiatives of sustainable development with strong regional appeal and respect to the traditions and identities of its people in its various cultural manifestations [13,39,40,41].

It also highlights the important contribution of UGGp in territorial action and co-participatory management of diverse natural areas, where a set of strategies should be considered, emphasizing mitigatory actions for environmental impacts (monitoring and caretaking), actions to prevent natural disasters, training of technical staff and multiplication of knowledge and environmental education (geoeducation). It is also important to ensure the alignment of these strategies with environmental regulation bodies, which are responsible for the intervention and application of stringent measures in accordance with the laws of each country. Cases such as fossil traffic in the territory of the Araripe UGG, for example [41], and the improper exploitation of resources or environmental risks such as the disasters that occurred in Brumadinho and Mariana, in the State of Minas Gerais, in Brazil [42,43,44] extrapolate the actions of a geopark and are necessary interventions of the police and judicial bodies adhering to the mission of the UNESCO program.

### 1.2. Natural Trail Management and Development Opportunity at Araripe UGG

The Araripe UNESCO Global Geopark, founded in 2006 as the first geopark of the Americas, after four evaluations with the green seal, has established itself as one of the oldest and most important geopark programs in the world. Managed by the Regional University of Cariri (URCA), it has been contributing to sustainable regional development and the formation of intellectual capital of high level in the region, with several programs of extension scholarships and research and relevant actions in safeguarding the heritage. The international relevance of the geodiversity of the Araripe UGG is centered on the existence of the Araripe Sedimentary Basin, which has in its composition a geographical feature called Chapada do Araripe (Araripe Plateau). In these records, the Cretaceous formations stand out, which accumulate sui generis fossil materials of the most diverse groups of animals and plants, characterizing the site as a Fossil-Lagerstätten (both Konzentrat-Lagerstätten and Konservat-Lagerstätte) [41,45,46]. Especially in geopark territories, geotourism, as a niche of tourism, focuses on the valuation and promotion of landscapes and geopatrimony in natural and urban environments, as attractions with significant potential for territorial development [46,47]. In this context, the Araripe UGG, due to its location and geographical characterization, has an intimate connection with the Chapada and the Araripe National Forest [48,49,50,51,52,53,54,55,56,57,58,59] and, given its landscape, naturalness and scenic beauty, presents relevant potential and opportunity for interaction and ecosystem health from the practice of green exercise as a scheduled or incidental activity [11,12,13,18,26,60,61,62,63,64,65,66,67].

The development of this study in Araripe is justified due to its wide circuit of natural trails arranged in the region and that present themselves as a unique alternative of intangible provisions and active visitation experiences focused on active and healthy lifestyles. Seeing, hearing, smelling, interacting and experiencing the territory sensorially stimulates the visitor at different times of the year where one has a unique experience when crossing the same trail in Araripe [12,13,14,27]. In this context, the importance of nature trails is emphasized as the main (or only) access route that allows the visitor to have contact with the ecosystem and its cultural appeal. Moreover, the management of a trail’s itinerary, besides allowing access, generates direct and indirect income opportunities for entrepreneurs, artisans and local stakeholders, such as tour guides and drivers of traditional or native communities, such as quilombolas, indigenous and riverine [12,22,23,24,25,26,62,66,68,69,70,71,72,73,74,75].

In the development of a proposed natural trails route or circuit, it is important to emphasize the adequate management of visitation impacts with a view to a sustainable exploitation of the territory, identifying the profile of visitation and the implications of its consequences, in order to establish priority actions to mitigate the impacts and manage the visitation sites. The management strategies of the impacts and the carrying capacity should guide the decision of managers, in order to stabilize the consequences and implications of the exploitation of natural resources and guide what is the necessary gap that allows the restoration and re-establishment of the biostasis of the ecosystem or the consequences may be potentially irreversible [13,27,68,76]. Regarding the accessibility of a trail, it is essential that the hiker has information before starting his adventure. Indicators that provide information on the available resources and opportunities, difficulty levels, safety aspects and exposure to various overloads, among others, are crucial in deciding on the crossing of a trail, which justifies this study. The definition of these indicators, besides allowing users to be adequately prepared, facilitates the good performance of several local professionals, such as tour guides, educators, sport and health professionals, among others. The data available on the trails also allow the practice of various adventure sports as an active experience of visitation correlated to the natural heritage [12,13,66,77,78,79].

The results generated from this study guide important actions and public health policies in order to consolidate green areas accessibility to the public, with information related to the health opportunities of ecosystems available in the region and correlated to environmental conservation and sustainable tourism development [9,12,13,22,26,28,61,62,63,64,65,66,67,68,69,70,71,72,73,74,75,76,77,78,79].

This study aims to discuss and contribute to these aspects of territorial enhancement contextualized as the UNESCO Global Geoparks program (UGGp) with a focus on sustainable regional development and green economy opportunities in the management of a territory. In this context, the objective of this research is, under the perspective of networks, to identify the centrality and connection indicators to classify the effort index and biomechanical overload of the nature trails of Sítio Fundão, Cachoeira da Missão Velha and Pontal de Santa Cruz, all located in geosites of UGG Araripe.

## 2. Materials and Methods

This was a documental [80,81] and exploratory study with transversal cut, quantitative approach to the data, developed on an ecological scale [81]. The universe of study was the UNESCO Global Geoparks Program and the locus was the Araripe Geopark as a case study. The documentary data of the Araripe UGG were: the Trails Signaling Plan, the Strategic Planning, the Master Plan and the periodic reports of the Geoconservation and Geotourism sectors approved in the revalidation of the last cycle in 2019 UNESCO [13,27,67,82,83,84]. The field studies were carried out between March 2019 and December 2021, with suspensions of activity due to accessibility restrictions because of the pandemic. The data were tabulated from field spreadsheets and the use of georeferencing and audiovisual equipment and applications: GPS, smartphones, tablets and digital and action cameras.

### 2.1. Study Locus and Inclusion Criteria—Trails of the Batateiras (Sítio Fundão Trail), Missão Velha Waterfall and Pontal de Santa Cruz Geosites

**Figure 1:**
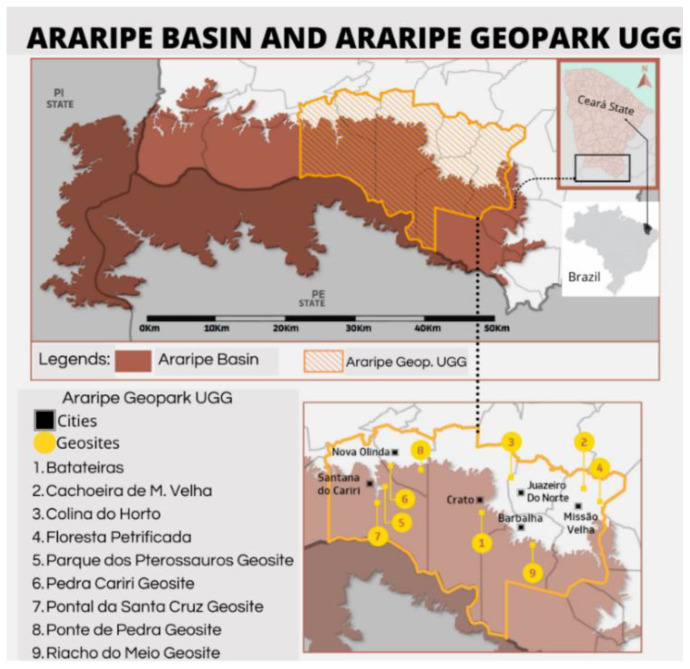
Map of the Territory and Geosites of Araripe UGG. Source: Research Collection.

The Araripe UNESCO Global Geopark (UGG), the focus of this research, is located in northeastern Brazil, in the extreme south of Ceará State, in the Cariri Cearense region, about 500 km from Fortaleza, the state capital. The first geopark of Brazil and the Americas, it is in an area of 3789 km^2^ after the state resizing and was approved in the last UNESCO certification in 2019. Its territory is spread over six municipalities: Crato, Juazeiro do Norte, Barbalha, Missão Velha, Nova Olinda and Santana do Cariri. It has nine geosites (Figure 1) open to the public for visitation, with relevant geological, paleontological, archaeological and historical content, allied to a valuable cultural heritage (see Figure 1). These geosites and the Araripe UGG territory have significant potential and opportunities for diverse green exercise practices [13,27,67,83,84].

As for the inclusion criteria, five of the nine geosites of the Araripe UGG present georeferenced trails and are promoted as attractions for visitation; the others (four) only have access trails [13,27]. Considering that the results of this study are a substantial part of a larger study already under development, we consider in this sample three geosites arranged in official publications of the Araripe UGG, these being: Batateiras Geosite (Sitio Fundão Trail) in the city of Crato, Missão Velha Waterfall Geosite in the city of Missão Velha and Pontal de Santa Cruz Geosite in the city of Santana do Cariri [13,27,67].

#### 2.1.1. Sítio Fundão Trail—Batateiras Geosite

**Figure 2:**
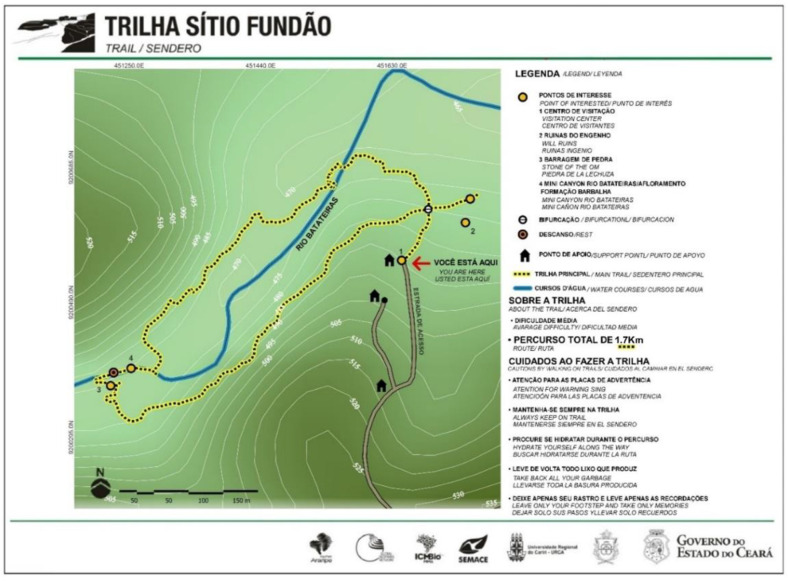
Sítio Fundão Trail in Batateiras Geosite. Source: Trail Signaling Plan Araripe UGG [82], adapted from research.

The trail of Sítio Fundão in Batateiras Geosite (Figure 2) is located in the geosite closest to the administrative headquarters of the Araripe UGG and the city center of Crato, georeferenced at south latitude 7°14′00.7″ S and west longitude 39°26′19.6″ W [67] (see Figure 1 and Figure 2). The geosite overlaps with a State Conservation Unit, given the record of its faunal and floristic importance, and its location at the foot of the Chapada do Araripe (Araripe Plateau) is in the middle of the urban area of the municipality. According to control data from the Secretariat of Environment of Ceará (SEMA), which is responsible for managing the area, the geosite/park has been gradually increasing its number of visitors [27]. Regarding the impacts of visitation, there have been increasing issues with the improper disposal of garbage and damage to infrastructure such as the protective railings that limit the area of the geosite. Despite this, the geosite is classified as a non-vulnerable area and the trail has been receiving maintenance and infrastructure improvement in order to optimize the visitor experience [27].

#### 2.1.2. Missão Velha Waterfall Geosite Trail

**Figure 3:**
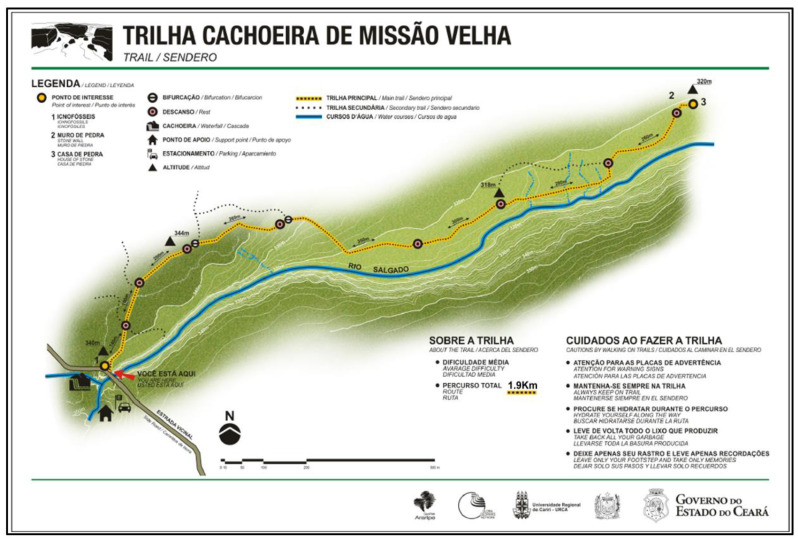
Missão Velha Waterfall Trail. Source: Trail Signaling Plan Araripe UGG [82].

The geosite trail, located in the Missão Velha Waterfall in the homonymous city, is situated in the waterfall site (see Figure 3), 3 km from the municipal seat at 7°13′21.5″ S latitude south and 39°08′36.8″ W longitude west, adjacent to the CE 153 highway [27,67,82]. The geosite is in the Municipal Park (Law no 002/02, Complementary Law no 017/02) and an area of the Natural Monument Rio Salgado Waterfall (Decree no 28.506/06) (see Figure 1 and Figure 3). It is characterized by waterfalls, approximately 12 m high, formed by the Salgado River with higher volumes in the rainy season between January and April [27,67]. The geosite receives about 1100 visitors per year with higher incidence of visitors during the highest waterfall flow; these numbers are underestimated due to the absence of a more reliable record of visitors [27]. The main impacts resulting from visitation are depredatory, especially the improper disposal of garbage, damage to signs, buildings, infrastructure, graffiti and vandalism to the geological heritage. Mainly due to the absence of a permanent management team at the geosite, it appears as a vulnerable area in the Matrix of Management Priority and Visitation Impacts of the Araripe UGG with recommendation of priority management action [27].

#### 2.1.3. Pontal da Santa Cruz Geosite Trail

**Figure 4:**
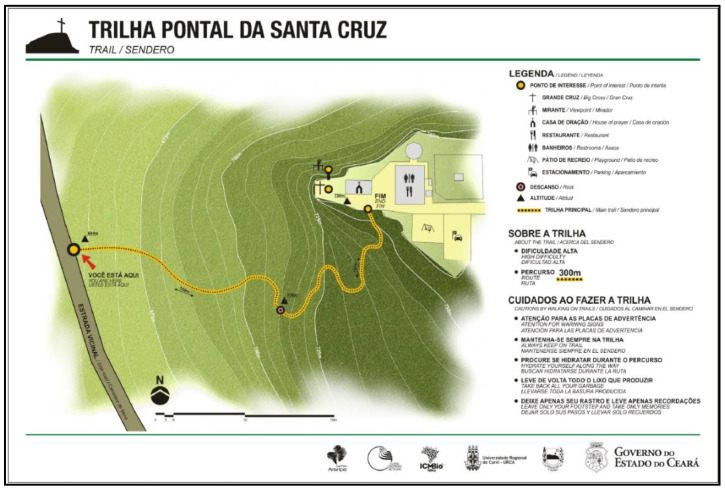
Pontal da Santa Cruz Geosite Trail. Source: Trail Signaling Plan Araripe UGG [82].

The Pontal de Santa Cruz Geosite Trail (Figure 4), in the community of the same name, is located 4 km from the city of Santana do Cariri with south latitude at 7°12′38.2″ S and west longitude at 39°44′04.7″ W (see Figure 1 and Figure 4) [27,67,82]. The trail provides access to the geosite that is located at the top of the Chapada do Araripe (Araripe Plateau), associated with the occurrence of the Exu Formation. The trail runs along the slope towards the top, and although it is a short stretch, it presents a staircase about 300 m long and reveals considerable altimetric variations [27,67].

The geosite receives about 32 thousand visitors per year, especially because it is part of the itinerary of the Plácido Cidade Nuvens Paleontology Museum, about 3 km from the geosite. In relation to the impacts of visitation, the geosite appears to be in a non-vulnerable situation and the trail has been receiving maintenance and infrastructure improvement in order to optimize the trail’s access [27].

The main impacts are related to inadequate waste disposal and damage to infrastructure such as railings and containment chains, evident along the trail. Even so, of the three geosites, it has the least visitation impacts, classified in blue in the Araripe UGG Management Priority Matrix. Such results are mainly because of the permanent presence of the management team, which inhibits depredatory actions and conducts periodic cleaning [27].

### 2.2. Analisys Instruments

To collect and treat the diversity of data and their multidisciplinary specificities in this investigation, we considered the review and integrative interpretation of methodologies and instruments already referenced and contextualized to the object of this study.

We highlight as a theoretical reference the application model proposed by Guimarães et al., (2021) [13] based on the conceptual proposal of Gabriel et al., (2018) [12]. The authors used a methodology for complex systems presented the Ecosystem Health Provision Spectrum (EHPS) based on a network of connections between the health potentials (EHP) and opportunities (EHO) in the investigated natural area. The model presents the results of the relationship between exposure variables in an ecological dimension considering aspects of geodiversity, biodiversity, climatic and meteorological diversity, aquatic diversity and trail classification as Ecosystem Health Potentials (EHP); and also presents aspects inherent to infrastructure and well-being experiences with nature [14] as Ecosystem Health Opportunities (EHO) [12,13].

The results of the Ecosystem Health Potentials and Opportunities of the investigated trails were regrouped and used as a primary matrix in the psychometric characterization of the indicators of severity of the environment, route orientation and terrain conditions from a rereading of the proposal of the Brazilian Association of Technical Standards (ABNT) in the Brazilian Standards (NBR) for tourism with hiking activities and trails classification (ABNT/NBR 15505-2:2008) [13,85,86], based on the Método para Lá Informacion de Excursiones—MIDE [87]. The standard considers aspects of environment severity, orientation, terrain characteristic and effort index. NBR 15505.2 is intended for the classification of trails without overnight stay and directed to non-athlete adults with light luggage [13,85].

In complement to NBR 15502.2 we propose the analysis of the biomechanical overload as a complementary and discriminant variable to the effort index by time. The biomechanical overload analysis aims to detect the behavioral trends of maximum plantar pressure values (MaxP) as risk factors for foot decompression during walking on various slopes [65,77,78]. To determine the biomechanical overload in human bipedal locomotion, we consider the reactive bearing force, plantar pressure, joint variation in the foot–ankle set (especially uphill) and knee (especially downhill), and the internal forces in these joints to identify the potential for overloading in exposure to the severity of the environment and the predisposition to an injury as a result of the change in gait pattern when traversing a trail [65,77,78,88,89,90,91,92]. The indicators for determining biomechanical overload are obtained from the linear regression and arithmetic mean of five indicators, based on the coefficient of determination and regression equation (R2 > 0.8) between the gradient and the representative values of MaxP [65,77,78]. In this investigation we considered the results attributed to biomechanical overload of the knee given the difference when compared to the absolute distance of the investigated trail.

### 2.3. Mathematical Modeling for Defining Total Travel Time

**Figure 5:**
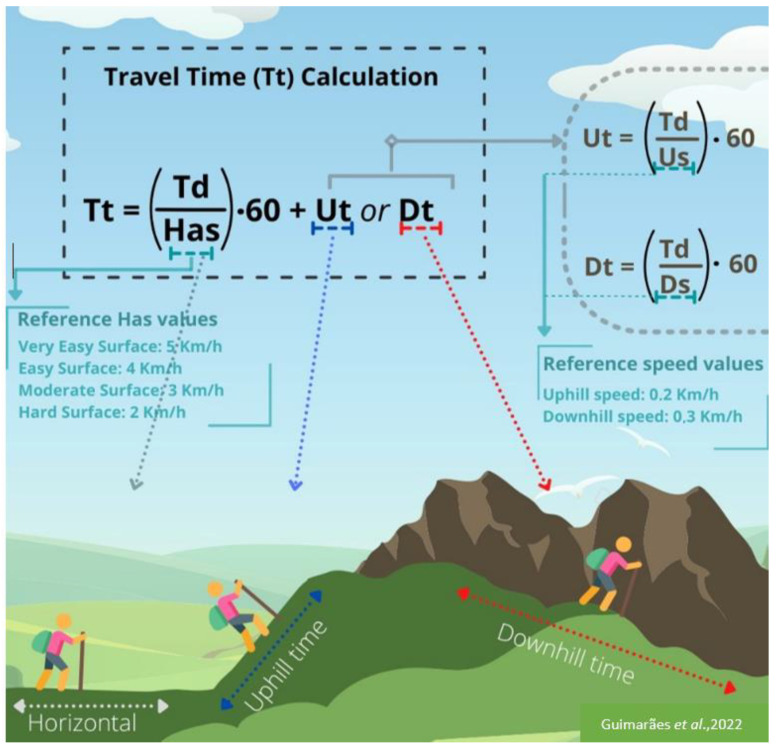
Schematic summary for calculating the Travel Time (Tt). Legend: Td: Traveled Distance; Has: Horizontal Average Speed; Ut: Uphill Time; Dt: Downhill Time; Us: Uphill Speed; Ds: Downhill Speed. Source: Research Collection [13].

The Travel Time (Tt) is proposed as an estimate that considers the division ratio between the Traveled Distance (Td) and the average speed on a horizontal route (Has), equivalent to a constant defined according to the difficulty attributed to the surface [13,85], multiplied by 60 s (for time in minutes). If there are sections of unevenness, the resulting value must be added to the Uphill or Downhill Traveled Time slope (Ut or Dt), the latter obtained from the division ratio between the Traveled Distance (Td) per section and the Uphill or Downhill Speed (Us or Ds), equivalent to a defined constant depending on the case [13,85], multiplied by 60 s (for time in seconds) (see Figure 5). It should be noted that if the stretch has no unevenness it is not necessary to calculate the speed of uphill and downhill. In this way, the general formula is:

$$Tt=\left(\frac{Td}{Has}\right)\cdot 60+Ut\;or\;Dt$$

Additionally, the complementary calculation of the uphill or downhill travel time is as follows:

$$Ut=\left(\frac{Td}{Us}\right)\cdot 60\;\mathrm{or}\;Dt=\left(\frac{Td}{Ds}\right)\cdot 60\;$$

### 2.4. Statistical Procedures

Network analysis is a contemporary statistical method that allows the evaluation of the structure and dynamics of a complex system. In the present study, the Effort Index and Biomechanical Overload is related to a series of several of different scales. In this sense, the analysis allows capturing the dynamic and non-linear nature of these relationships, and in this way it is possible to calculate which variables have the greatest impact on the evaluated system. Therefore, it is possible to plan possible interventions based on the most central variables [93]. The network analysis was used to formulate the network pattern and the indicators of centrality and connection in order to determine the overload of exposure in the classification of natural trails. The strength centrality indicators were reported. The strength indicator is essential to understand which variables present the most robust connections in the current network pattern [93].

The “Fruchterman–Reingold” algorithm was applied; therefore, data were shown in the relative space in which variables with stronger permanent statistics were together and those with less strongly applied variations repelled one another [94]. To improve the accuracy of the network we used the model “random fields of pair wise Markov”. The algorithm adds an “L1” (regularized neighborhood regression) penalty. The regulation is estimated by a less complete selection and contraction operator (LASSO) that controls the sparse network [95]. The extended Bayesian information criterion (EBIC) to select the Lambda of the regularization parameter was observed. EBIC uses a hyperparameter (y) that determines how much EBIC selects sparse models [96]. For a better visualization of the matrix, the network is presented in a graph that includes the variables (nodes) and the relations (lines). The blue color represents positive associations, and the red color represents negative ones. The thickness and intensity of the colors represent the magnitude of the associations. The qgraph package of RStudio was used [93,94].

## 3. Results

### 3.1. Characterization Regarding the Ecosystem Health Potential and Opportunity

#### 3.1.1. Geodiversity and Biodiversity

**Figure 6:**
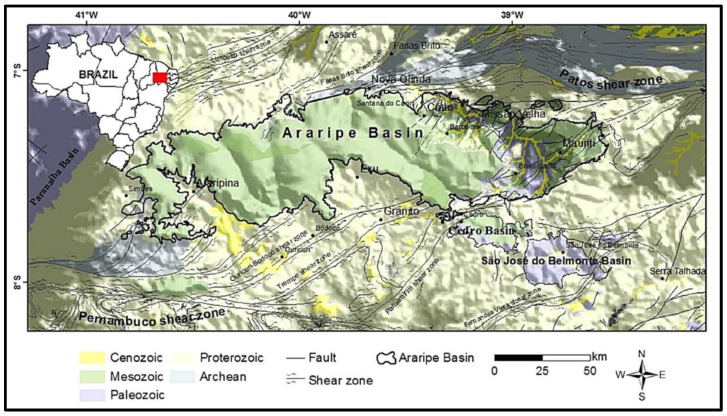
Simplified geological map of the Araripe Basin. Source: Miranda et al. 2014 [103].

The influence of the elements of geodiversity and biodiversity are evident in the classification of trails [12,13], starting from the premise that the genesis of the sites is directly related to geological and biogeographic aspects [97,98,99,100]. Thus, one can understand that the pre-established relief forms derive from a set of actions conditioned by endogenous dynamics (greater power of action at the subsurface level) and exogenous dynamics (greater power of action at the surface). In the case of this study, the occurrence of the Araripe Basin [45,67,100,101,102] stands out as fundamental in the process of defining these aspects (see Figure 6).

Similarly, the development of soils maintains a connection with the source rock, whether this is the underlying or a rock different from the local system, in the case of transported material. This directly influences the type of predominant vegetation, and together with other aspects such as climate and topography will constitute the main parameters in the evolution of the local environment, including defining the physiognomy [103,104].

In the analysis of the existing trails in the Araripe UGG, one realizes that the occurrence of major geographical accidents in the middle of the morphoclimatic domain of Caatinga, such as the Chapada do Araripe, creates the most sui generis aspects [13,53,104].

In this context, the classifications of the Pontal da Santa Cruz Geosite Trail and the Sitio Fundão Trail will be presented. As a counterpoint, the Missão Velha Waterfall trail was analyzed, as it does not belong to the Chapada do Araripe area [104].

#### 3.1.2. Sítio Fundão Trail in Batateiras Geosite

**Figure 7:**
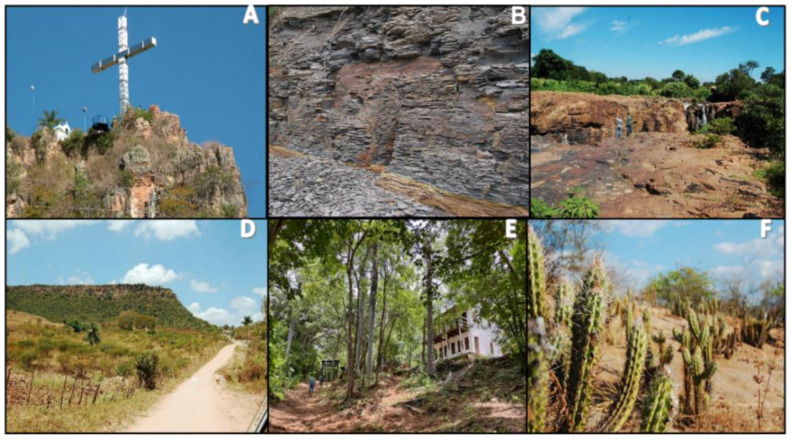
Geodiversity and associated biodiversity of Araripe UGG (**A**): Geosite Pontal da Santa Cruz, with emphasis on the rocky outcrop of the Exu Formation. (**B**): Batateiras Geosite, in the homonymous riverbed, with outcrop of the Batateira Layers. (**C**): Missão Velha Waterfall Geosite, with emphasis on the Cariri Formation. (**D**): Geosite Pontal da Santa Cruz, vegetation associated with the Caatinga and Carrasco biomes. (**E**): Geosite Batateiras, vegetation associated with the Caatinga biome already signaling transition to Cerradão. (**F**): Geosite Missão Velha Waterfall, vegetation associated with the Caatinga biome in its most apparent physiognomy in the “sertão”. Source: Research Collection.

Considering the territory data, the biodiversity of the Sítio Fundão trail in Batateiras Geosite, based on the Shannon–Weaver index, was 4.65, indicating 84% prevalence of the 5.5 reference indicator for tropical forests, which indicates that the studied area has a high floristic diversity (see Figure 7E) [49,50,105,106,107]. The trail clearly shows a phytophysiognomic differentiation of the Caatinga biome, being an area with characteristics of open ombrophylous forest and deciduous seasonal/semideciduous forest [53,106]. In a transitional way, it is possible to observe elements of forested savannah (cerradão).

Considering the geodiversity values, the Sítio Fundão trail and Batateiras geosite present 1115 scores, corresponding to 280 scores of scientific value, 200 scores of degradation risk, 335 scores of educational value and 300 scores of tourist value [108], indicating 69.7% prevalence of the reference value of 1600 scores [13]. The whole circuit is associated with the occurrence of the Barbalha Formation [102]. In terms of geodiversity, it is the post-rift record of the Araripe Basin, or the period of plate cooling and consequent thickening of the lithosphere. We highlight the occurrence of the Batateira Layers which represents the first lacustrine configuration characterized by anoxia and preservation of organic materials (see Figure 7B) [101,102,109,110]. There are records of ostracods and coprolites among the blackened shales.

The trail extends over more stable and flat areas, with stony stretches of rocks transported by the semi-active drainage channels, which require a little more from the visitors. Because this is an area related to the middle aquifer of the Araripe Basin complex, with the existence of the Batateiras River, there are stretches of wet walkways, both on the banks and in the crossing of the body of water. These stretches require more care by visitors, as they are made of smooth ground, due to the presence of clays and the formation of mud mats. The stretches vary according to the time of the year, with dominance increasing during the rainy season and decreasing during the dryer seasons.

#### 3.1.3. Mission Velha Waterfall Trail

On the Missão Velha Waterfall trail, the Shannon–Weaver index of biodiversity indicated 3.6 scores, reaching 64% of the reference indicator (5.5 scores) [105]. Outside the Chapada do Araripe domain, the site predominantly reflects the classic northeastern semi-arid area and its exclusive biome: the Caatinga [53,106,107,111]. The soil is typically sandy with the presence of low and sparse bushy vegetation, generally cactaceous xerophytes (see Figure 7F). One can also observe the presence of medium-sized deciduous species, and even larger ones that have adapted to the dry climate, such as the Yellow Ipê (*Handroanthus albus*) and the Juazeiro (*Ziziphus joazeiro*) [53]. The trail is predominantly flat, and the biggest challenge is the adaptation of the visitor to the very hot and dry climate, although there is the possibility of resting near a natural miner (Fonte do Pinga) and at the end of the trail (historical ruins) [27,67].

Considering the geodiversity values, the Missão Velha Waterfall trail and its homonymous geosite present 1090 scores, corresponding to 190 scores of scientific value, 295 scores of degradation risk, 335 scores of educational value and 270 scores of touristic value [108], indicating 68.15% of prevalence of the 1600 scores reference value [13]. The trail runs parallel to the course of the Salgado River associated with the Cariri Formation, which is unique in the Siluro-Ordovician sequence of the Araripe Basin (Paleozoic). It is basically immature, medium to very coarse grained sandstone formations, typical of a paleoenvironment dominated by a dynamic of intertwined rivers (see Figure 7C) [102,112]. There are records of ichnofossils and tracks of indeterminate invertebrates; however, so far these are without stratigraphic value [113].

#### 3.1.4. Pontal de Santa Cruz Trail

The Pontal de Santa Cruz trail presented 2.9 scores in the Biodiversity Index (Shannon–Weaver), representing 54% of the reference value of 5.5 scores [105]. Along the trail there is a tropical xeromorphic Subcaducifolia forest, notably sustained by the condition of the plateau, with the predominance of the Carrasco and Caatinga vegetation (see Figure 7D) [53,106,107,111,114]. In the lower areas of the surrounding valley, one can notice the relation of these domains with the geomorphologic variations existing from the top of the Chapada do Araripe to the access to the valleys.

Considering the geodiversity values, the Pontal de Santa Cruz geosite trail presents 1075 scores, corresponding to 190 scores of scientific value, 225 scores of degradation risk, 340 scores of educational value and 320 scores of tourist value [108], indicating 67.19% of prevalence of the 1600 scores reference value [13]. The sandstone rocks of the Exu Formation are characterized by being the most recent deposition of the Araripe Basin (Albian-Cenomanian interval) outcrop in this area (see Figure 7A) [102]. These sandstones are very permeable, and were fundamental in the genesis of the upper aquifer of the basin, ensuring the collection of water in the rainy seasons that contribute to the recharge of springs and water sources.

The path has been improved for the better performance of the tour by visitors, including with more safety. These interventions harmonize with the landscape composition, and do not compromise the natural dynamics of the hillside environment. Some adaptations even make use of the existing sandstone outcroppings, such as the support rings, for use in the most vertical part of the trail.

### 3.2. Aquatic Diversity, Climatic and Meteorological Exposure

In order to consider aquatic diversity, we follow the classification as indicators considering the potential salutogenic effects of water access in different representations in Table 1 [12,13,19,115,116,117].

The values obtained by the salutogenic indicators of aquatic diversity refer to the geosite 11 scores equivalent to 73% of prevalence of the reference value of 15 scores. The Batateiras River flows through the Batateiras Geosite (Sítio Fundão trail). Although it is perennial, its volume is seasonal, varying between the rainy season and in the dry season. The interaction is limited and depends on the time of year. Especially in periods of higher volume, bathing in the riverbed is common on the part of visitors and bathing is acceptable. Even so, it is noteworthy for the risk of accidents on the slippery rocks and also flash flood, which have previously taken victims. As for potability, there is an absence of this information from the responsible bodies, and for this reason we recommend purification before consumption.

In the Missão Velha Waterfall trail the prevalence was 53.3% based on the 8 scores obtained in the classification of the salutogenic indicators. At the Missão Velha Waterfall Geosite the presence of water is perennial and, given the characteristic of the Araripe UGG territory, it also undergoes alteration as to its volume from its waterfalls fed by the Salgado River. It is noteworthy that the interaction is indirect because there is no access from the trail to the riverbed; although there is a staircase for technical access in the canyons we do not consider this as a visitor attraction due to its limitations. Although some visitors bathe in the waters of the waterfall, there is no access described as offering a visitation experience, due to the risk of accidents and falls into the void in the canyons area. For this reason, bathing is improper/prohibited. It stands out due to a water fountain that spouts from the ground called “Fonte do Pinga” in the final stretch of the trail. As for potability, there are no data from the responsible bodies, but there are signs of discharges of waste in the tributaries, so it is not recommended for consumption.

In the Pontal de Santa Cruz trail, the result in the salutogenic classification of the aquatic diversity indicated 5 scores equivalent to 33.3% of prevalence of the reference used. On the Pontal de Sta. Cruz trail there is no presence of natural water sources of any nature as far as interaction is concerned. However, there is restaurant infrastructure at the end of the trail allowing access to drinking water from the public water supply system and also bottled mineral water for consumption upon purchase.

In the characterization regarding climate and meteorological exposure on the trails we considered the reference of the historical series on the territory of the Araripe UGG [13,117] and classified this based on the proposed methodology that considers the aspects of climate and green tunnels present in the studied trails (see Table 2).

The territory of the Araripe UGG is intrinsically conditioned by the Chapada do Araripe and its interaction with air masses. The territory is characterized as a humid and sub-humid tropical climate, influenced by the morphoclimatic domain of the Caatinga (northeastern semi-arid) [53,106,111] and with irregular rainfall given the concentration of rainfall in cycle one, between the months of December and April. The periods of greater exposure to extreme weather are arranged in cycles 3 and 4 (from August to November), characterized mainly by high temperatures, low relative humidity and the greatest incidence of sunlight [13,117,118,119]. The climate characterization and seasonality have a direct influence on the vegetation, arrangement and supply of green tunnels on the trails and its relevant impact on environmental thermoregulation due to the phytophysiological processes (see Table 2) [12,13,19,120,121,122,123]. The results show that the green tunnel indicator proves to be the most discriminating regarding exposure and climatic conditions in the crossing of a nature trail (letter “f” highlighted in Table 2) [13]. We considered and ranked the seasonal cycles from lowest to highest exposure based on climatic and meteorological constraints and their influence on landscape elements and ecosystem characteristics. The exposure and severity of the environment for green exercise was considered as an indicator in order to determine the effort overload of the hiker.

**Figure 8:**
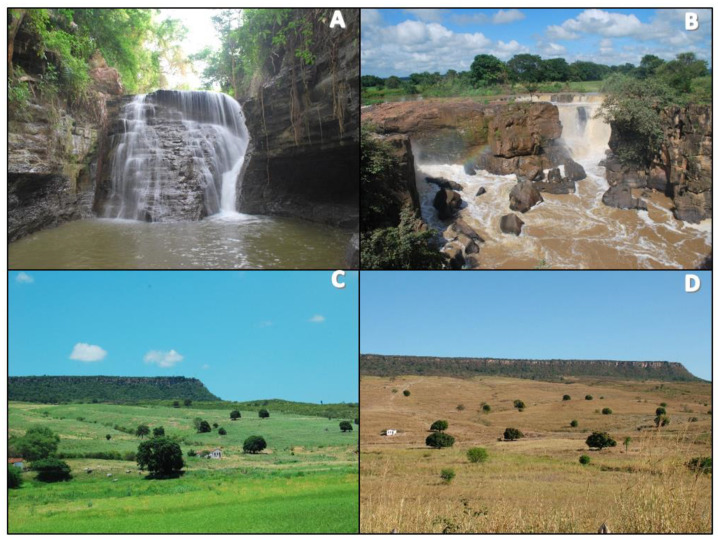
Water availability and climatic aspects. (**A**)—Batateiras geosite (Sítio Fundão trail), waterfall of the homonymous river. (**B**)—Missão Velha Waterfall geosite, canyon of the Salgado River. (**C**) and (**D**)—Pontal da Santa Cruz geosite, landscape differences related to the predominance of different seasons (rainy and dry); Araripe UGG. Source: Research Collection.

In this context, the Sitio Fundão trail in Batateiras geosite showed the highest total score with 49 scores and 68% prevalence of the reference indicator of 72 scores. The indicators show that the trail is less influenced by climatic factors specifically due to the higher incidence of green tunnels during the year given the characterization of the vegetation in the biome (see Figure 8A). On the other hand, both the Missão Velha Waterfall (see Figure 8B) and Pontal de Santa Cruz trails (see Figure 8C,D), with 46 accumulated scores and 64%, present more seasonal influence given the predisposition in the ecosystem of the Caatinga vegetation and all the conditions of exposure of the dynamic landscape evident in cycle 3 and 4 from August to November, including a drier period and more extreme exposure to meteorological conditions (see Table 2).

### 3.3. Infrastructure and Visiting Experience

As for infrastructure, the Batateiras geosite in the Sítio Fundão Park, the location of the investigated trail, obtained 6 scores reaching 100% prevalence of the indicator. It is noteworthy that the site is managed by the Secretary of Environment of Ceará (SEMA) with technical support from the Araripe UGG [27]. The site has a parking lot, sentry box and entrance gate, technical office for management and visitor support. As for the attractions, we highlight a geodesy, a two-story taipa house (made of wood and clay), the ruins of a sugar cane mill gear from 1880 (which suffered a fire in 2019) and ruins of buildings of an old hydroelectric plant from 1939. The site is cut by the Batateiras River and the intermittent springs there are full of indigenous legends and stories, the most famous being the legend of the “Pedra da Batateira” (Batateira stone) [27,67]. There is a diversity of trail circuits, including a small trail designed for the visually impaired called “Trilha dos Sentidos” (Trail of the Senses). A wheelchair adapted for trails (Jullieti chair) is also available at the site [13]. The site is monitored by security guards and also has a team of environmental technicians and guides who work on a daily basis. Visitation and access to the park is regulated, allowing access preferably by appointment [13,27].

The Missão Velha Waterfall geosite, with the homonymous trail, obtained 2 scores and 33.3% of the infrastructure prevalence indicator (6 scores), the lowest score among the trails and geosites studied [27]. The site is managed by the Municipality of Missão Velha and with technical support from the Araripe UGG. The site has great historical relevance, with traces of prehistoric indigenous populations and its role as a meeting point of cangaceiros in the early twentieth century. On the trail are preserved ichnofossils, traces of activity of ancient organisms (aquatic invertebrates) with a vermiform appearance. There is the presence of geoforms characterized as “marmitas”, circular cavities excavated by abrasion in circular water percolation movements that form swirls, that are often considered as aspects of geomorphological heritage [124,125]. At the end of the trail there are ruins of stone houses from the first colonizations in Cariri in the 17th century. Despite being a place that always attracts visitors, especially in the period of flooding and late afternoon because of the beautiful sunsets, it requires more investment as observed by the indicator obtained. We highlight the need for improvements in support and security infrastructure, in environmental technical staff and tourist reception. The management priority matrix studies classify the geosite in a vulnerable situation and indicate the need for a revitalization program for the area, which is in the process of being feasible with the purpose of recovering the waterfall’s balneability and increasing the infrastructure for visitation [13,27].

The Pontal de Santa Cruz Geosite and the trail that receives its name obtained 6 scores for infrastructure and, like the Batateiras geosite, reached the maximum score in the prevalence of the indicator [27]. The trail is located at one of the extremes of the Chapada do Araripe at an altitude of about 750 m, and along the steep trail of projected steps 300 m long it is possible to gradually see the immensity of the Cariús river valley and the plateau. At the end of the trail the visitor can enjoy a panoramic viewpoint, a bucolic chapel and two monumental crucifixes, one made of wood and the other of metal. The visitor also has a circuit of figures that tell the legend of the “haunting” of Pontal that only ceased to haunt the place after a procession of the residents who raised the large wooden crucifix to bless the village. The place also has a family restaurant with regional cuisine, a children’s playground, wooden benches for resting and contemplation in addition to the sale of historical and sacred souvenirs alluding to Miss Benigna of Santana do Cariri, beatified by the Vatican in 2022 and Father Cícero Romão, considered a popular saint who receives thousands of faithful devotees at the Colina do Horto Geosite in the Araripe UGG territory [13,27,67].

Considering the intangible and salutogenic benefits of ecosystem health services, including the multiple opportunities and sensory experiences in the transposition of nature trails, we consider the following results [12,13,14]. The Sítio Fundão trail obtained 33 scores equivalent to 55% of the indicator of prevalence used (60 scores), the Missão Velha waterfall trail obtained 36 scores with 60% of prevalence and the trail of Pontal de Santa Cruz trail presented 27 scores representing 45% of prevalence of the maximum score (see Table 3). It is noteworthy that the 60 scores used as totality refer to the listed offers of adventure activities throughout the Brazilian territory by official entities of the segment [13].

The descriptive data and characterization of ecosystem health potentials and opportunities were used as the matrix for psychometric categorization regarding the severity of the environment (see Table 4), route orientation (see Table 5) surface and ground type (see Table 6).

**Table 4:** Psychometric Rating of the Severity of the Environment: Sítio Fundão, Missão Velha Waterfall and Pontal de Sta. Cruz trails; Araripe UGG.

Slightly Severe (SS)	Moderately Severe (MS)	Severe (S)	Quite Severe (QS)	Very Severe (VS)			
Up to 3 Scores	4 to 5 Scores	6 to 8 Scores	9 to 12 Scores	Starting at 13 Scores			
Route Detailing					SF	MVW	PSC
1. Ecosystem Health Potential (EHP)—Geodiversity and Biodiversity							
(a) exposure to spontaneous detachments of stones during the course;							✔
(b) exposure to stone detachments caused by the group itself or another during the course;							✔
(c) possibility of falling into the void or down a steep slope;						✔	✔
(d) possibility of the ground or floor giving way inferring a fall into the void, water, caves, etc.							
(e) the existence of passages where it is necessary to use the hands to progress along the course;							✔
(f) existence of narrow passages or entrance in holes, caves and caverns							
(g) path through dense vegetation or through irregular terrain that may make it difficult to orient or locate people in any part of the path					✔		
(h) exposure to and risk with potentially poisonous local fauna: swarms of bees, spiders, snakes, small reptiles or amphibians, etc;					✔	✔	✔
(i) exposure to and risk with potentially aggressive local fauna; large animals: buffalo, cattle, wolves, foxes, jaguars, alligators, piranhas etc;					✔	✔	✔
2. Ecosystem Health Potential (EHP)—Aquatic Diversity							
(j) exposure to permanently slippery, rocky or flooded stretches during the course;					✔		✔
(k) crossing rivers or other bodies of water with currents, by ford (without bridge);					✔		
(l) risk of flash floods, floods, sudden variation of tides, undulations and strong current					✔		
(m) region or stretches without access to potable water.						✔	
3. Ecosystem Health Potential (EHP)—Climate and Weather Exposure							
(n) exposure to slippery or flooded stretches due to rain during the route;					✔		✔
(o) high probability of heavy or continuous rainfall for the period;					✔		
(p) high probability that during the night the temperature may drop below 0 °C;							
(q) high probability that the temperature drops below 5 °C and the relative humidity exceeds 90%;							
(r) high probability of exposure to strong or cold winds							
(s) high probability that the relative humidity is below 30					✔	✔	✔
(t) high probability of exposure to heat at temperatures above 32 °C;					✔	✔	✔
(u) long stretches of exposure to strong sunlight;					✔	✔	✔
(v) eventual decrease of visibility due to atmospheric phenomena that may increase considerably the difficulty of orientation or the location of people in any part of the route;							
4. Ecosystem Health Opportunity (EHO)—Infrastructure and Visiting Experience							
(w) time to complete the activity equal to or greater than 1 h of marching without passing an inhabited place, an emergency telephone (or cellular or radio signal) or an open road with vehicle flow; another hiker, group, or technical personnel.							
(x) time of performing the activity equal or superior to 2 h of march without passing by an inhabited place, an emergency telephone (or cellular or radio signal) or an open road with vehicle flow; without meeting another hiker, group, or technical personnel.							
(y) time to complete the activity equal or superior to 3 h of walking without passing by an inhabited place, an emergency telephone (or cellular or radio signal) or an open road with vehicle flow; without meeting another hiker, group, or technical personnel.							
(z) the difference between the time needed to complete the route and the amount of remaining daylight hours at the end of the day (available at the time of year considered) is less than 3 h.							
RESULTS					11 pts (QS)	7 pts (S)	11pts (QS)

Legend: SF: Sitio Fundão; MVW: Missão Velha Waterfall; PSC: Pontal de Santa Cruz. SOURCE: ABNT—NBR 15505-2:2008 [85] and Guimaraes et al. 2021 [13], adapted for research.

**Table 5:** Classification according to course orientation. Sítio Fundão, Missão Velha Waterfall and Pontal de Santa Cruz trails; Araripe UGG.

No	Reference	Detailing	SF	MVW	PSC
1	Well-defined paths and intersections	Well-marked or signposted main paths, with clear intersections with explicit or implicit indication. Staying on the path requires no effort to identify the route. Occasionally, it may be necessary to follow a line marked by an unmistakable geographic accident (for example, a beach or the shore of a lake) (see Figure 9A,B).		✔	✔
2	Path or signage that indicates continuity	There is a clear path delineation on the terrain or signage for the continuity of the route. It requires attention to the continuity and intersection of other paths, but without the need for precise interpretation of landmarks. This condition applies to most signalized paths that use, on the same route, different types of paths with numerous crossings, e.g., motor vehicle tracks, footpaths, riding paths and fields marked by (well-located and well-maintained) milestones (see Figure 9C).	✔		
3	It requires the identification of landmarks and cardinal points	Even if the itinerary is developed by tracing over trails, routes marked by landmarks (rivers, valley bottoms, coastlines, ridges, rocky coasts, among others), or marks of other people’s passing, the choice of the appropriate route depends on the recognition of the landmarks and cardinal points.			
4	Requires off-track navigation skills	There is no tracing over the terrain, no security of relying on landmarks on the horizon. The route depends on understanding the terrain and plotting the course.			
5	Requires navigation to use alternative, previously unknown routes	The route depends on understanding the terrain and route design, and requires navigational skills to complete the route. The route’s course may be unexpectedly interrupted by obstacles that need to be avoided.			
	RESULTS		**2**	**1**	**1**

Legend: SF: Sitio Fundão; MVW: Missão Velha Waterfall; PSC: Pontal de Santa Cruz. Source: ABNT—NBR 15505-2:2008 [85], adapted for research.

**Table 6:** Classification according to surface and ground conditions.

No	Course	Detailing	SF	MV	PSC
1	Traveling on Flat Surfaces	Roads and vehicle lanes, regardless of their slope. Stepped roads with level and even pavement. Beaches (sand or gravel) with level and firm ground			
2	Pathfinding No obstacles	Routes through various firm terrains, but which maintain the evenness of the ground, well-marked trails that do not have large inclines or obstacles that require great physical effort to overcome. Routes through uniform terrain such as fields and pastures that are not too steep (see Figure 10A)		✔	
3	Traveling over graded trails or uneven terrain	Travel on trails with obstacles or uneven steps of different size, height and slope. Off-trail walking over uneven terrain. Crossings through stony areas or areas with rocky outcrops (stone slabs). Stretches of loose rock, unstable quarries, very exposed roots, gravel or large erosions (see Figure 10B)	✔		
4	Obstacle course	Paths with obstacles that may require jumping or using the hands up to I Sup. (UIAA grade for climbing or vertical progression) (see Figure 10C)			✔
5	Route that requires vertical techniques	Sections that require climbing techniques from Grade II to III Sup. (UIAA grade for climbing or vertical progression). Requires the use of specific equipment and techniques. The existence of these sections is subject to mention in the section “Specific Conditions”, according to Annex B [85].			
	Results		3	2	4

Legend: SF: Sitio Fundão; MV: Missão Velha; PSC: Pontal de Santa Cruz. Source: ABNT—NBR 15505-2:2008 [85]. Adapted for research.

**Figure 9:**
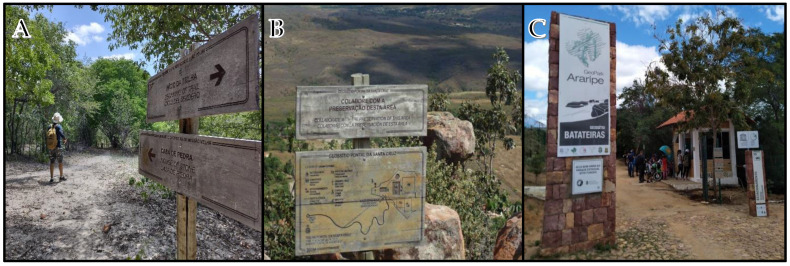
Course and Orientation: Signs along the trails (example). (**A**)—Missão Velha Waterfall trail; (**B**)—Pontal da Santa Cruz trail; (**C**)—Sítio Fundão trail (Batateiras Geosite); Araripe UGG. Source: Research Collection.

**Figure 10:**
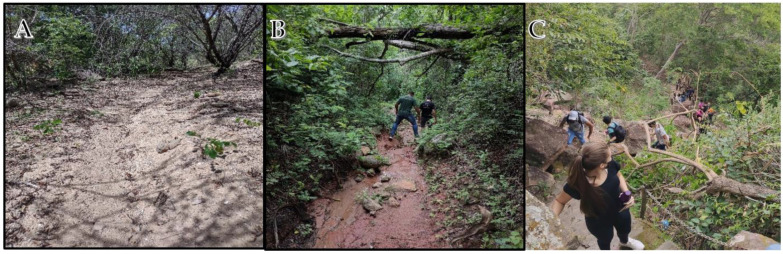
Surface ground and altimetric variations. (**A**)—Missão Velha Waterfall Trail: predominantly flat altimetry, regular surface with burlap, sandy and stable floor; (**B**)—Sítio Fundão Trail: predominantly irregular altimetry, little unevenness, surface of the with burlap, stony floor, with waterlogged and slippery stretches; (**C**)—Pontal da Santa Cruz Trail: predominantly steep altimetry, irregular surfaces with burlap, stretches with stones and stable projected rocky floor (steps); Araripe UGG. Source: Research Collection.

### 3.4. Mathematical Model Application

The indicators used for the Classification of the Effort Index of the trails follow the following methodological script: (a) definition of the distance of the trail; (b) stratification of the distance of the stretches based on the positive and negative altimetric variation (slopes and inclinations); (c) characterization of the surface and ground (Very Easy, Easy, Moderate, Difficult) (see Table 6 and Figure 10); (d) definition of the average speed on the horizontal based on the previous classification: 5 km/h Very Easy Surface, 4 km/h Easy Surface, 3 km/h Moderate Surface, 2 km/h Difficult Surface; (e) application of speed correction considering positive or negative gradient: 0.2 km/h on the uphill and 0.3 km/h on the downhill. Based on these indicators and from the mathematical modeling proposed in this study, the time (per route) and absolute (total) to cross the path is defined (see Table 7 as an example) [13,85]. Such information, inherent to the difficulties of the path, is relevant to the decision of the hiker to walk the trail.

**Figure 11:**
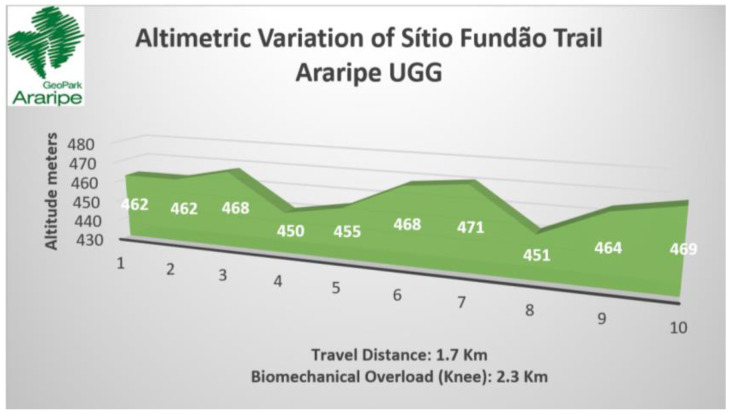
Altimetric Variation of Sítio Fundão trail; Araripe UGG. Source: Research Collection.

The Sítio Fundão trail has a distance of 1.7 km and presents 52.3 m of accumulated gradient (positive) and 38 m of accumulated slope (negative), characterized as a trail with predominantly positive gradient (see Table 7). The trail has a circular characteristic with entry and exit in the same point but with different routes and paths or signage that indicates continuity (see Table 5 and Figure 9C). As for the surface and ground, they are classified as difficult, characterized as rocky, unstable, muddy, slippery and wet, which justifies the walking speed of 2 km/h on most of the trail (see Table 6 and Figure 10B). Considering the biomechanical overload of the knee, the course is equivalent to a horizontal trail of about 2.3 km (see Figure 11).

**Figure 12:**
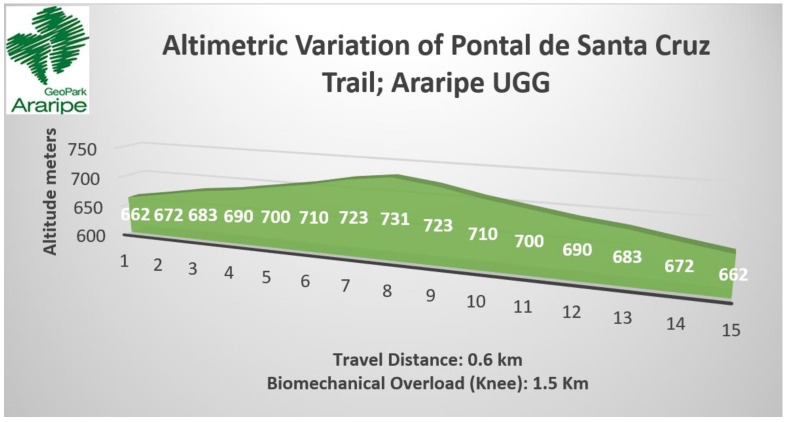
Altimetric Variation of Pontal de Santa Cruz trail; Araripe UGG.

The Pontal de Santa Cruz trail has a distance of 0.6 km with 81 m of accumulated gradient (positive) and 81 m of accumulated slope (negative) (see Table 8). The trail starts and ends in the same point with well-defined paths and intersections (see Table 5 and Figure 9B). As for the surface and ground, it is classified as difficult, highlighted by long staircases and sections with mandatory use of hands for grade I climbing, which justifies the 2 km/h travel speed (see Table 6 and Figure 10C). Considering the biomechanical overload of the knee, the route is equivalent to a horizontal trail of about 1.53 km (see Figure 12).

**Figure 13:**
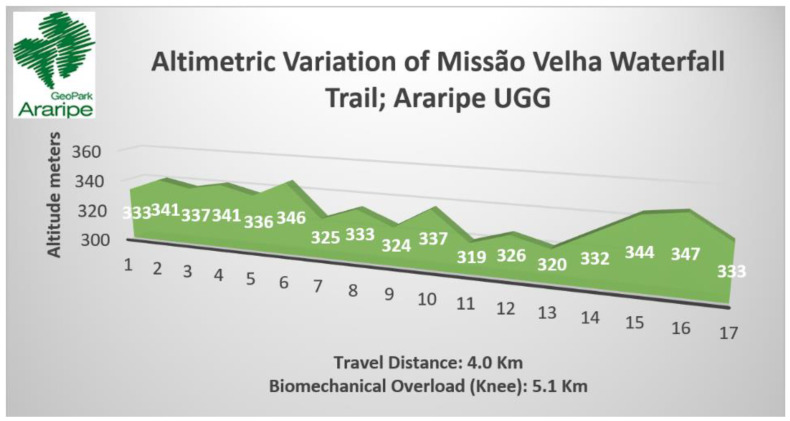
Altimetric Variation of Missão Velha Waterfall trail; Araripe UGG. Source: Research Collection.

The 4.0 km long Missão Velha Waterfall trail has 77 m of accumulated slope (positive) and 77 m of accumulated slope (negative) (see Table 9) with entry and exit in the same point with well-defined paths and intersections (see Table 5 and Figure 9A). As for the surface and ground, this is classified as moderate with little altimetric variation, stable floor and some stretches with slightly unstable sandy floor that justifies the average 3 km/h of travel (see Table 6 and Figure 10A). Considering the biomechanical overload of the knee, the course is equivalent to a horizontal trail of about 5.1 km (see Figure 13).

Based on the indicators of altimetric variation (positive and negative) and the distance per stretch, we estimated the biomechanical overload based on the maximum values of plantar pressure with emphasis on the biomechanical load on the knee that was calculated using only the MaxFcomRF data considering the plantar support zone T2-5 and the levels of femoral patella compression. The accumulated load at the end of five thousand horizontal steps (approximately 5 km) was considered as a reference to determine the gain relative to the absolute distance of the walk.

The results obtained considered two variables related to the biomechanical overload (5 and 7), the first related to the additional travel distance of the trail and the second related to the time to traverse the trail, using, as reference, the average horizontal velocity (Has) on the additional route and the absolute travel distance. The results of each of the seven indicators were tabulated in order to determine the point prevalence as the primary matrix to feed the network analysis (see Table 10). Considering the variables of biomechanical overload in the knee and the additional travel time, we highlight the highest levels of the effort index in the Pontal de Santa Cruz (+139.4%), Sítio Fundão (+29.9%) and Missão Velha Waterfall (+22.4%) trails, respectively.

The result of the prevalence of the variables arranged in Table 10 served as the primary matrix for network analysis. In order to fulfill the objective proposed in this study of classifying the effort index and the exposure burden to ecosystem health potentials and opportunities, we considered the correlation between the selected indicators.

### 3.5. Network Analysis of Variables

In Table 11 we present the result of the correlation between the variables of the indicators that underlie the network structure (mention factors) and their arrangement of the investigated trails. Among the indicators used we highlight the emerging pattern arranged in each of the networks presented.

**Figure 14:**
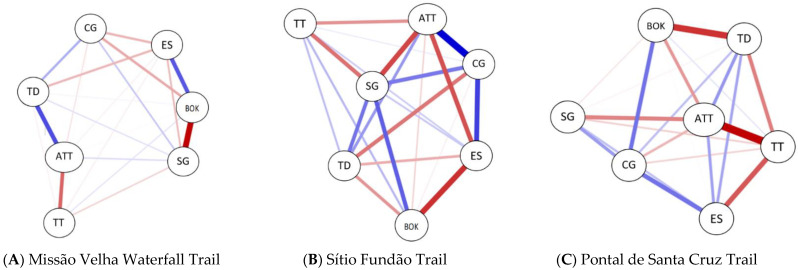
Network analysis of variables of the Sitio Fundão, Missão Velha and Pontal de Santa Cruz trails; Araripe UGG. Legend: 1: ES: Environmental Severity; 2: CG: Course Guidance; 3: SG: Surface and Ground; 4: TD: Travel Distance; 5: BOK: Biomechanical Overload of the Knee; 6: TT: Travel Time (NBR.15505.2); 7: ATT: Additional Travel Time. 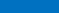 Positive Associations 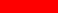 Negative Associations.

In the network results in Figure 14, the blue color represents the positive associations and the red color represents the negative associations. The thickness and intensity of the colors represent the magnitude (strength) of the associations. For the results we focused on mainly analyzing the power of connection as an indicator of centrality in order to determine which indicators are more prevalent in defining the overload of effort presented in the ecosystem.

In the A network (see Figure 14A)—Missão Velha Waterfall trail, we find that the most influential centrality indicators for the integrity of the network were indicator 6 (negative) regarding the total time established based on NBR 15505.2; indicator 5 (positive) on the biomechanical overload on the knee; indicator 3 (positive) regarding the terrain and indicator 2 (negative) regarding the orientation.

In the B network (see Figure 14B)—the trail of Sítio Fundão in Batateiras Geosite, we find that indicator 7 (positive), relating to the additional time of biomechanical overload, indicator 6 (negative) relating to the total time based on NBR 15505.2, indicator 3 (positive) regarding the terrain and indicator 5 (negative) in relation to biomechanical overload in the knee are the most influential indicators.

In the C network (see Figure 14C) Pontal de Santa Cruz trail, the same indicators of Sítio Fundão trail were presented as central to the difference between the indicators presented in the B and C network, which were in relation to the type of positive or negative associations between the variables. Therefore, we highlight that indicator 7 (positive) is related to the additional time of biomechanical overload, indicator 6 (positive) is related to the total time based on NBR 15505.2, indicator 3 (positive) is in regard to the terrain and indicator 5 (negative) relates to the biomechanical overload on the knee.

## 4. Discussion

On the Pontal de Santa Cruz trail, there is a strong negative connection between indicator 7, related to biomechanical overload, the additional time and indicator 6, related to the expected time because of the distance and speed of travel (based on the ground surface). Although the stretch has a total distance of 600 m, the effort index to which the hiker is submitted due to the biomechanical overload in the knee (evidenced in the altimetry and accentuated unevenness) is 2.6 times greater, equivalent to a horizontal course of 1527 m increasing considerably the time to finish the course. In this sense, the biomechanical overload due to altimetric variation presents itself as a determining indicator in the effort index. Another indicator that stood out in the network was 3, regarding the terrain surface, which besides presenting a long and steep staircase, was classified as a trail with obstacles that may require jumping or the use of hands for climbing or vertical progression (level I). The trail in question, in its final sections, has rings embedded in the rocks to support the hands and overcome obstacles, a feature that increases the degree of difficulty. Even when considering the accumulated time of the absolute distance of the trail plus the complementary distance of the biomechanical overload on the knee, the trail is classified as low effort, with an estimated travel time of up to 1 h. In this sense the classification only by travel time may underestimate the demands that the hiker may encounter along the way.

On the Mission Velha Waterfall trail, the most evident indicator of effort index was indicator 5, regarding the biomechanical overload on the knee due to the increase in travel time that considers aspects related to distance, travel speed based on the surface of the terrain and the altimetry presented. An inherent characteristic of this trail is that the biomechanical overload shows a negative relationship with indicator 3 regarding the surface of the terrain (or ground), characterized as stretches with firm and regular surfaces that do not present obstacles and sharp gradients that require great physical effort to be overcome. In this sense the biomechanical overload on the knee is evidenced not by the difficulty attributed to the conditions of the unevenness or floor, but rather as a result of the increase in the course time by about 22.4%, exposing the hiker to a horizontal trail equivalent to 5100 m despite the 4000 m presented on the course. Even with the increase in additional travel time due to the biomechanical overload, the trail scored 2 scores and was classified with a moderate effort index given the travel time of 1 to 3 h.

In the Sítio Fundão trail, to define the indicators of effort index, we highlight in the network the strong connection presented by indicator 7 related to biomechanical overload and increased additional time of the route of about 30% exposing the hiker to a biomechanical overload in the knees to a horizontal trail equivalent to 2320 m despite the 1700 m presented in the route. Another indicator that deserves attention is number 3, related to the terrain surface. On the trail, there are stretches with obstacles due to biotic material such as burlap, exposed roots, trunks and branches, of different size, height and slope. The trail passes through irregular terrain, crosses stony areas, rocky outcrops, riverbeds and slippery ground. The trail in question does not present a relevant altitude variation that has greater weight in the attribution of the knee overload and the compensatory time to traverse the trail. It is noteworthy that on the trail studied, the increase in time and exposure to biomechanical overload is characterized in particular by constraints regarding the ground surface and risk of injury to the foot and joint groupings with emphasis on the tibio-tarsal joint as a result of walking on an unstable surface. Considering the time for classification of the effort index of the trail, the additional travel time increased the score and the classification from easy with 1 score (up to 1 h of travel time) to moderate with 2 scores (from 1 h to 3 h of travel time).

The previous information, related to the conditions of the trails in an ecological dimension, proved to be fundamental in order to prepare the hiker for the experience as to the effort index along the path, both for the more vigorous and planned exercise or incidental physical activity, with contemplative purpose, for example [12,13,60].

The results presented in the networks highlighted the indicators biomechanical overload as variables with greater comparative centrality and connection strength. The combinations of the two methodologies showed relevant internal consistency considering that the data collected are correlated and meet both methods, with emphasis on the positive and negative gradients and the travel distance. The NBR-15505.2 standard [85] presents as a final product the displacement time for classification of the effort index, while the biomechanical overload considers the exposure of contractile and non-contractile tissues and the response of multiple structures of the lower limbs, with emphasis on the foot and knee joint, and from the regression modeling presents as a result an estimated compensation in relation to the distance of the horizontal course that translates into an increase in displacement time predicted in the NBR 15505.2 standard. In this sense, the primary data from the NBR 15505.2 standard feed the matrix for analysis of the biomechanical overload that presents itself as an indicator with a relevant degree of discrimination in relation to time in the definition of the effort index, as presented in the results of the network analysis of this research. It is noteworthy that the cut-off used to define the effort index based on travel time requires revisions in order to include and trails shorter than 5 km. Shorter routes have a strong inclusive potential as incidental physical activity (as a means) in active, healthy and sustainable daily mobility, such as pavements and walking paths, bicycle lanes, green corridors and tunnels, and especially in parks and natural areas within urban environments. The classification methodologies of trails in general are directed to trails that require a certain complexity of organization and logistics for access and enjoyment by the population and that, probably, will not be components of a programmed and daily physical activity, but rather as attractions for visitation as a result of a tourist or adventure route. It is with this justification that this research, despite consideration, did not emphasize the travel time for the definition of the effort index in fact, since the trails described here are of short lengths and, therefore, there is little discrimination considering the travel time predicted in different methodologies [13,65,66,77,78,79,85,86,87,88,89].

Although the methodologies used to define the effort index and biomechanical overload are convergent, we highlight as a limitation of this study the need to treat the data separately; the use of a single modeling would optimize data processing. In this sense, we recommend for future research the combination of mathematical modeling presented in this study (see Figure 5) with the regression modeling used for biomechanical overload [77,78]. Another relevant point is the possibility of readjustment of the horizontal average speed (Has); we consider in this study trails from 2 to 5 km/h (based on the ground surface) for walking, but that can be adjusted for running at higher speeds (above 5 km/h). However, since the study was limited to the maximum values of plantar pressure in human gait, we recommend future research regarding biomechanical overload that substantiates the changes in average speed based on slopes by adjusting these variables for mechanized (mountain bike for example), equestrian or motorized displacements and adapting the mathematical modeling proposed in this study (see Figure 5).

Finally, it is recommended of studies for the development of mobile applications and software that optimize data collection and evaluations in the field, which would contribute significantly to technological innovation and decision-making in the management of areas of interest based on the diagnosis and definition of the baselines of the territories. Despite the ecological dimension of this research, the data described here can be deepened with research on the human dimension—considering the use of gadgets and feasible technologies to collect indicators that consider the physiological response of effort in exposure to green exercise.

## 5. Conclusions

The focus of this research was to contribute a methodology for the classification of the effort index and biomechanical overload that subsidized the development of a circuit of natural trails in the territory of Araripe UGG. For this, the potentials and opportunities of ecosystem health services, the experience of well-being and the salutogenic benefits of green exercise were characterized. From this perspective, the aim was to inform the hikers about the experience of completing the trails studied here.

The primary indicators of the trails classified in this study considered aspects of geodiversity, biodiversity, climatic characterization, aquatic diversity, surface and terrain conditions, orientation profile, well-being and visitation experience. The analysis procedures used in the treatment of these indicators sought to minimize subjectivity in the evaluation of the indices of effort, overload and visitation experience resulting from the transposition of a nature trail.

One of the main challenges of this research was to establish a positive correlation between the multiple ecosystem variables and consolidate a proposal that had a relevant potential applicability in the evaluation of a territory. In the network analysis, the data were treated from a non-linear perspective and, despite being adequate to the investigation, challenging characteristics were evident during the treatment and consolidation of the results. In this sense, we emphasize the analysis instruments, as theoretical references, and the psychometric characterization used to define the prevalence matrix of the indicators that fed the network analysis as the statistical procedure used. We highlight as an innovation of this research the review and presentation of the mathematical modeling to define the travel time from the ecological variables (see Figure 5). By inserting the psychometric scores of the variables in the equation, the final product is the travel time of the evaluated trail. The time to cross a trail, as proposed in this study, presents itself as an indicator of decisive effort for the hiker and the proposition of classification methodologies in order to reduce subjectivity in the definition of this variable are always challenging.

The methodology presented here was developed in order to make feasible the application of a simplified and replicable modeling in the management of nature trails. We understand that optimizing the management of territories and regions (especially those with fewer resources) is essential in order to consolidate a network of nature trails aggregated to a high tourist, scientific, educational, cultural and welfare potential, in order to promote sustainable opportunities in geopark territories and in diverse natural areas. The methodological procedural presented here, as well as those of the visitation impact matrix [27] and ecosystem health services [13], were consolidated in the management of the Araripe UGG, and evaluated and approved during the revalidation of the UNESCO green seal in the 2019 cycle. Such methodologies are already used in the management of the territory and in the training of the technical team, undergraduate and postgraduate academics, tour guides, hikers, researchers and in geoconservation, geotourism and geoeducation programs.

The methodological innovations, in the light of scientific rigor, added value and optimized the management and the opportunities offered by the Araripe UGG territory, in addition to contributing to the formation of high-level intellectual capital to act in the region, fostering income generation, conserving heritage and ecosystems and contributing to a sustainable relationship between man and the environment as aims of the UNESCO Global Geoparks program.

## Figures and Tables

**Table 1:** Diversity and aquatic interaction, Batateiras, Missão Velha Waterfall and Pontal de Sta. Cruz, Araripe UGG.

Indicators	Batateiras	Missão Velha W.	Pontal de Santa Cruz
(a) Availability	3	3	2
(b) Interaction	2	1	0
(c) Bathing	2	1	0
(d) Potability	2	2	3
(e) Risk	2	1	0
Total	11 scores	8 scores	5 scores

Subtitle: (a) Availability: 1 Inaccessible; 2 Seasonal; 3 Perennial. (b) Interaction: 1 Indirect: see, hear, smell; 2 Direct: consumption, bathing; 3 Diverse: swimming, fishing; boating. (c) Bathing: 1 forbidden/improper; 2 satisfactory; 3 good/excellent. (d) Potability: 1 not applicable; 2 requires purification; 3 potable. (e) Risk: 1 high; 2 medium; 3 low. Source: Guimarães et al. 2021 [13]; adapted for research.

**Table 2:** Classification of Weather and Climate Exposure in the Araripe Trails UGG.

Trails	Sítio Fundão				Missão Velha W.				Pontal de Santa Cruz			
Exposure Indicator	Seasonal Cycle				Seasonal Cycle				Seasonal Cycle			
	I	II	III	IV	I	II	III	IV	I	II	III	IV
(a) Insolation	3	2	1	1	3	2	1	1	3	2	1	1
(b) Temperature	2	3	1	1	2	3	1	1	2	3	1	1
(c) Rainfall	3	2	1	2	3	2	1	2	3	2	1	2
(d) Relative Humidity	3	2	1	2	3	2	1	2	3	2	1	2
(e) Winds	1	2	3	2	1	2	3	2	1	2	3	2
(f) Green Tunnel	3	3	2	2	3	2	1	2	3	2	1	2
Seasonal by Cycle Score	15	15	9	10	15	13	8	10	15	13	8	10
Total Scores	49 scores				46 scores				46 scores			

Subtitle: Seasonal Cycle I: December to April; II: May to July; III: August to September, IV: October to November. (a) Insolation: 3 Ideal, 2 Medium, 1 Worst; (b) Temperature: 3 Ideal, 2 Medium, 1 Worst; (c) Rainfall: 3 Ideal, 2 Medium, 1 Worse; (d) Relative Humidity: 3 Optimal, 2 Medium, 1 Worst; (e) Winds: 3 Ideal, 2 Medium, 1 Worst; (f) Green Tunnel: 3 Ideal, 2 Medium, 1pt. Worst. Source: Guimarães et al. 2021 [13], adapted for research.

**Table 3:** Wellness Experiences and Health Opportunities; Sítio Fundão, Missão Velha Waterfall; Pontal de Santa Cruz trails; Araripe UGG.

Experience	Sítio Fundão Trail	Missão Velha Waterfall Trail	Pontal de Santa Cruz Trail
Nature Visions, excerpts with:	1. Trees and vegetation of various colors; 2. Geoforms; 3. Cultural elements; 4. Ruins; 5. Viewpoints 6. Rivers, 7. Water fountains; 8. Various Birds; 9. Wild animals.	1. Trees and vegetation of various colors; 2. Geoforms; 3. Cultural elements; 4. Ichnofossils; 5. Waterfalls, 6. Rivers; 7. Water springs; 8. Canyons; 9. Ruins; 10. Various birds; 11. Wild animals	1. Trees and vegetation of various colorations; 2. Geoforms; 3. Cultural elements; 4. Lookouts; 5. Mountains and Valleys; 6. Various Birds; 7. Wild Animals
Sounds in the Environment, excerpts with:	1. Various songs of birds; 2. Insects; 3. Running water; 4. Waterfalls; 5. Voice; 6. Music; 7. Sound of wind; 8. Silence	1. Various songs of birds; 2. Insects; 3. Running water; 4. Waterfall; 5. Voice; 6. Music; 7. Silence; 8. Cattle mooing; 9. Sound of wind.	1. Various songs of birds; 2. Insects; 3. Running water; 4. Voice; 5. Silence; 6. Sound of wind;
Odors in the Environment, excerpts with:	1. Wetland; 2. Native fruits; 3. Vegetation and flowers; 4. Wild animals	1. Wetland; 2. Native fruits; 3. Vegetation and flowers; 4. Wild animals; 5. Cattle	1. Vegetation and Flowers; 2. Native fruits; 3. Wild animals; 4. Regional cuisine
Interaction and Opportunity	1. Flora; 2. Bird Watching; Trail: 3. Walking; 4. Trail Run; 5. Mountain Biking; 6. Meditation; 7. Waterscape, 8. Camping; 9. Study; 10. Research Interaction with: 7. Hikers (alone) or 8. Groups.	1. Flora; 2. Bird Watching; Trail: 3. Walk; 4. Trail run; 5. Mountain Biking, 6. Meditation; 7. Waterscape, 8. study, 9. Research Interaction with: 10. Hikers (alone) 11. Groups.	1. Flora; 2. Bird Watching; Trail: 3. Walking; 4. Trail run 5. Rappelling; 6. Meditation 7. Study, 8. Research Interaction with: 9. Hikers (alone) or 10. Groups.
Total Score	33 scores	36 scores	27 scores

Source: GUIMARÃES et al. (2021) [13] apud RUSSEL et al. (2013) [14]; adapted for research.

**Table 7:** Effort Index Classification by route and total of the Sítio Fundão trail; Araripe UGG.

Route	Altitude	TD	SG–Has (2, 3, 4, 5)	Speed: Uphill—Downhill		
Start	462 m	Km	2 km/h (Hard)	0.2 km/h US/0.3 km/h DS	Formula	Tt
Route 1	462 m	0.1 km	2 km/h	0.2 km/h US	$Tt=\left(\frac{Td}{Has}\right)\cdot 60+\left(\frac{Td}{Us}\right)\cdot 60$	3 min 30 s
Route 2	468 m	0.1 km	2 km/h	0.2 km/h US	$Tt=\left(\frac{Td}{Has}\right)\cdot 60+\left(\frac{Td}{Us}\right)\cdot 60$	3 min 30 s
Route 3	450 m	0.2 km	2 km/h	0.3 km/h DS	$Tt=\left(\frac{Td}{Has}\right)\cdot 60+\left(\frac{Td}{Ds}\right)\cdot 60$	6 min 40 s
Route 4	455 m	0.1 km	2 km/h	0.2 km/h US	$Tt=\left(\frac{Td}{Has}\right)\cdot 60+\left(\frac{Td}{Us}\right)\cdot 60$	3 min 30 s
Route 5	468 m	0.1 km	2 km/h	0.2 km/h US	$Tt=\left(\frac{Td}{Has}\right)\cdot 60+\left(\frac{Td}{Us}\right)\cdot 60$	3 min 30 s
Route 6	471 m	0.1 km	2 km/h	0.2 km/h US	$Tt=\left(\frac{Td}{Has}\right)\cdot 60+\left(\frac{Td}{Us}\right)\cdot 60$	3 min 30 s
Route 7	451 m	0.3 km	2 km/h	0.3 km/h DS	$Tt=\left(\frac{Td}{Has}\right)\cdot 60+\left(\frac{Td}{Ds}\right)\cdot 60$	10 min
Route 8	464 m	0.4 km	2 km/h	0.2 km/h US	$Tt=\left(\frac{Td}{Has}\right)\cdot 60+\left(\frac{Td}{Us}\right)\cdot 60$	14 min
Route 9	469 m	0.2 km	2 km/h	0.2 km/h US	$Tt=\left(\frac{Td}{Has}\right)\cdot 60+\left(\frac{Td}{Us}\right)\cdot 60$	7 min
Route 10	476 m	0.1 km	2 km/h	0.2 km/h US	$Tt=\left(\frac{Td}{Has}\right)\cdot 60+\left(\frac{Td}{Us}\right)\cdot 60$	3 min 30 s
TD		1.7 km		Effort Index: 2 scores;	Little Effort (up to 1 h)	58 min 40 s

Legend: TD: Travel Distance; SG: Surface and Ground; Has: Horizontal average speed (2 Hard, 3 Moderate, 4 Easy, 5 Very Easy); Us: Uphill speed; Ds: Downhill Speed; Tt: Travel Time. Effort Index: Little Effort (up to 1 h); Moderate (1 to 3 h); Significant (3 to 6 h); Intense (6 to 10 h); Extraordinary (over 10 h). Source: ABNT-NBR 15505.2 [85]. Adapted for research.

**Table 8:** Effort Index Classification by route and total of the Pontal de Sta. Cruz Trail; Araripe UGG.

Route	Altitude	TD	SG–Has (2, 3, 4, 5)	Speed: Uphill—Downhill		
Start	662 m	Km	2 km/h (Hard)	0.2 km/h US/0.3 km/h DS	Formula	Tt
Route 1	672 m	0.05 km	2 km/h	0.2 km/h US	$Tt=\left(\frac{Td}{Has}\right)\cdot 60+\left(\frac{Td}{Us}\right)\cdot 60$	1 min 45 s
Route 2	683 m	0.05 km	2 km/h	0.2 km/h US	$Tt=\left(\frac{Td}{Has}\right)\cdot 60+\left(\frac{Td}{Us}\right)\cdot 60$	1 min 45 s
Route 3	690 m	0.04 km	2 km/h	0.2 km/h US	$Tt=\left(\frac{Td}{Has}\right)\cdot 60+\left(\frac{Td}{Us}\right)\cdot 60$	1 min 24 s
Route 4	700 m	0.04 km	2 km/h	0.2 km/h US	$Tt=\left(\frac{Td}{Has}\right)\cdot 60+\left(\frac{Td}{Us}\right)\cdot 60$	1 min 24 s
Route 5	710 m	0.04 km	2 km/h	0.2 km/h US	$Tt=\left(\frac{Td}{Has}\right)\cdot 60+\left(\frac{Td}{Us}\right)\cdot 60$	1 min 24 s
Route 6	723 m	0.04 km	2 km/h	0.2 km/h US	$Tt=\left(\frac{Td}{Has}\right)\cdot 60+\left(\frac{Td}{Us}\right)\cdot 60$	1 min 24 s
Route 7	731 m	0.04 km	2 km/h	0.2 km/h US	$Tt=\left(\frac{Td}{Has}\right)\cdot 60+\left(\frac{Td}{Us}\right)\cdot 60$	1 min 24 s
Route 8	723 m	0.04 km	2 km/h	0.3 km/h DS	$Tt=\left(\frac{Td}{Has}\right)\cdot 60+\left(\frac{Td}{Ds}\right)\cdot 60\;$	1 min 20 s
Route 9	710 m	0.04 km	2 km/h	0.3 km/h DS	$Tt=\left(\frac{Td}{Has}\right)\cdot 60+\left(\frac{Td}{Ds}\right)\cdot 60$	1 min 20 s
Route 10	700 m	0.04 km	2 km/h	0.3 km/h DS	$Tt=\left(\frac{Td}{Has}\right)\cdot 60+\left(\frac{Td}{Ds}\right)\cdot 60$	1 min 20 s
Route 11	690 m	0.04 km	2 km/h	0.3 km/h DS	$Tt=\left(\frac{Td}{Has}\right)\cdot 60+\left(\frac{Td}{Ds}\right)\cdot 60$	1 min 20 s
Route 12	683 m	0.04 km	2 km/h	0.3 km/h DS	$Tt=\left(\frac{Td}{Has}\right)\cdot 60+\left(\frac{Td}{Ds}\right)\cdot 60$	1 min 20 s
Route 13	672 m	0.05 km	2 km/h	0.3 km/h DS	$Tt=\left(\frac{Td}{Has}\right)\cdot 60+\left(\frac{Td}{Ds}\right)\cdot 60$	1 min 40 s
Route 14	662 m	0.05 km	2 km/h	0.3 km/h DS	$Tt=\left(\frac{Td}{Has}\right)\cdot 60+\left(\frac{Td}{Ds}\right)\cdot 60$	1 min 40 s
TD		0.60 km		Effort Index: 2 scores;	Little Effort (up to 1 h)	20 min 30 s

Legend: TD: Travel Distance; SG: Surface and Ground; Has: Horizontal average speed (2 Hard, 3 Moderate, 4 Easy, 5 Very Easy); Us: Uphill speed; Ds: Downhill Speed; Tt: Travel Time. Effort Index: Little Effort (up to 1 h); Moderate (1 to 3 h); Significant (3 to 6 h); Intense (6 to 10 h); Extraordinary (over 10 h). Source: ABNT-NBR 15505.2 [85] adapted for research.

**Table 9:** Effort Index Classification by route and total of the Missão Velha Waterfall Trail; Araripe UGG.

Route	Altitude	TD	SG–Has (2, 3, 4, 5)	Speed: Uphill—Downhill		
Start	333 m	Km	3 km/h (Moder.)	0.2 km/h US/0.3 km/h DS	Formula	Tt
Route 1	341 m	0.1 km	3 km/h	0.2 km/h US	$Tt=\left(\frac{Td}{Has}\right)\cdot 60+\left(\frac{Td}{Us}\right)\cdot 60$	2 min 30 s
Route 2	337 m	0.05 km	3 km/h	0.3 km/h DS	$Tt=\left(\frac{Td}{Has}\right)\cdot 60+\left(\frac{Td}{Ds}\right)\cdot 60$	1 min 10 s
Route 3	341 m	0.05 km	3 km/h	0.2 km/h US	$Tt=\left(\frac{Td}{Has}\right)\cdot 60+\left(\frac{Td}{Us}\right)\cdot 60$	1 min 15 s
Route 4	336 m	0.3 km	3 km/h	0.3 km/h DS	$Tt=\left(\frac{Td}{Has}\right)\cdot 60+\left(\frac{Td}{Ds}\right)\cdot 60$	7 min
Route 5	346 m	0.2 km	3 km/h	0.2 km/h US	$Tt=\left(\frac{Td}{Has}\right)\cdot 60+\left(\frac{Td}{Us}\right)\cdot 60$	5 min
Route 6	325 m	0.4 km	3 km/h	0.3 km/h DS	$Tt=\left(\frac{Td}{Has}\right)\cdot 60+\left(\frac{Td}{Ds}\right)\cdot 60$	9 min 20 s
Route 7	333 m	0.6 km	3 km/h	0.2 km/h US	$Tt=\left(\frac{Td}{Has}\right)\cdot 60+\left(\frac{Td}{Us}\right)\cdot 60$	15 min
Route 8	324 m	0.1 km	3 km/h	0.3 km/h DS	$Tt=\left(\frac{Td}{Has}\right)\cdot 60+\left(\frac{Td}{Ds}\right)\cdot 60\;$	2 min 20 s
Route 9	337 m	0.3 km	3 km/h	0.2 km/h US	$Tt=\left(\frac{Td}{Has}\right)\cdot 60+\left(\frac{Td}{Ds}\right)\cdot 60$	7 min 30 s
Route 10	319 m	0.3 km	3 km/h	0.3 km/h DS	$Tt=\left(\frac{Td}{Has}\right)\cdot 60+\left(\frac{Td}{Ds}\right)\cdot 60\;$	7 min
Route 11	326 m	0.2 km	3 km/h	0.2 km/h US	$Tt=\left(\frac{Td}{Has}\right)\cdot 60+\left(\frac{Td}{Us}\right)\cdot 60\;$	5 min
Route 12	320 m	0.1 km	3 km/h	0.3 km/h DS	$Tt=\left(\frac{Td}{Has}\right)\cdot 60+\left(\frac{Td}{Ds}\right)\cdot 60\;$	2 min 20 s
Route 13	332 m	0.5 km	3 km/h	0.2 km/h US	$Tt=\left(\frac{Td}{Has}\right)\cdot 60+\left(\frac{Td}{Us}\right)\cdot 60\;$	11 min 15 s
Route 14	344 m	0.1 km	3 km/h	0.2 km/h US	$Tt=\left(\frac{Td}{Has}\right)\cdot 60+\left(\frac{Td}{Ds}\right)\cdot 60\;$	2 min 30 s
Route 15	347 m	0.2 km	3 km/h	0.2 km/h US	$Tt=\left(\frac{Td}{Has}\right)\cdot 60+\left(\frac{Td}{Us}\right)\cdot 60$	4 min 40 s
Route 16	333 m	0.5 km	3 km/h	0.3 km/h DS	$Tt=\left(\frac{Td}{Has}\right)\cdot 60+\left(\frac{Td}{Ds}\right)\cdot 60$	11 min 40 s
TD		4 km		Effort Index: 2 points	Moderate (1 to 3 h)	1 h 35 min 30 s

Legend: TD: Travel Distance; SG: Surface and Ground; Has: Horizontal average speed (2 Hard, 3 Moderate, 4 Easy, 5 Very Easy); Us: Uphill speed; Ds: Downhill Speed; Tt: Travel Time. Effort Index: Little Effort (up to 1 h); Moderate (1 to 3 h); Significant (3 to 6 h); Intense (6 to 10 h); Extraordinary (over 10 h). Source: ABNT-NBR 15505.2 [85]. Adapted for research.

**Table 10:** Prevalence Matrix of Variables for Network Analysis; Araripe UGG trails.

Reference	Cases (Tails)			Sample	Prevalence (%)		
VARIABLES	SF	MVW	PSC	Total	SF	MVW	PSC
1. Environmental Severity (ES)	11	7	11	26	42.3%	26.9%	42.3%
2. Course Guidance (CG)	2	1	1	5	40%	20%	20%
3. Surface and Ground (SG)	3	2	4	6	50%	30%	60%
4. Travel Distance (TD)	1.7 km	4 km	0.6 km	100%	100%	100%	100%
5. Biomechanical Overload of the Knee (BOK), distance considered	2.320 km	5.1 km	1.527 km	100%	+36.5%	+27.5%	+154.5%
6. Travel Time NBR.15505.2 (TT)	58 m 40 s	1 h 35 m	20 m 30 s	100%	100%	100%	100%
7. Additional Travel Time (ATT)	17 m 45 s	21 m 34 s	28 m 30 s	100%	+29.9%	+22.4%	+139.4%

Legend: SF: Sítio Fundão; MVW: Missão Velha Waterfall; PSC: Pontal de Santa Cruz.

**Table 11:** Centrality measures of variables to define the network modeling; Araripe UGG.

	Strength Centrality		
Variables	SF	MVW	PSC
1. Environmental Severity (ES)	0.420	0.053	0.233
2. Course Guidance (CG)	0.406	−0.799	−0.552
3. Surface and Ground (SG)	0.817	0.844	0.765
4. Travel Distance (TD) NBR 15505.2	−0.473	−0.214	0.233
5. Biomechanical Overload of the Knee (BOK)	−0.652	1.513	−0.552
6. Travel time NBR.15505.2 (TT)	−1.710	−1.516	0.765
7. Additional Travel Time (ATT)	1.192	0.117	1.474

Legend: SF: Sítio Fundão; MVW: Missão Velha Waterfall; PSC: Pontal de Santa Cruz.

## Data Availability

Not applicable.
